# Association between the Behavioral Activation Mechanism and Depression Severity: Focusing on Avoidance Patterns of University Students

**DOI:** 10.3390/bs14080713

**Published:** 2024-08-14

**Authors:** Koki Takagaki, Satoshi Yokoyama

**Affiliations:** 1 Health Service Center, Hiroshima University, 1-7-1 Kagamiyama, Higashihiroshima 739-8514, Japan; 2 Faculty of Humanities, Niigata University, 8050 Ikarashi 2-no-cho, Nishi-ku, Niigata 950-2181, Japan; syoko@human.niigata-u.ac.jp

**Keywords:** subthreshold depression, depression, avoidance, behavioral activation

## Abstract

Subthreshold depression is a risk factor for depression among university students. Positive environmental reinforcement is a significant mediator of avoidance and depression in healthy university students. However, this relationship is understudied in those with subthreshold depression or depression. Therefore, this study examined these associations in healthy university students and those who fit the criteria for subthreshold depression or depression. We conducted an online survey with 1200 undergraduate students (600 women and 600 men, mean age = 20.61, SD = 1.59). The results revealed significant differences in avoidance patterns (behavioral social, behavioral nonsocial, cognitive social, and cognitive nonsocial), with participants with depression presenting the highest scores. The results of the mediation analysis were similar to those of previous studies in healthy groups. However, in the subthreshold-depression group, positive environmental reinforcement was mediated by behavioral social and behavioral non-social avoidance and depressive symptoms. In the group with depression, positive environmental reinforcement was mediated only by behavioral nonsocial avoidance and depressive symptoms. Associations between avoidance patterns, positive environmental reinforcement, and depressive symptoms vary with the degree of depressive symptoms. The results revealed by this study provide new foundational insights to prevent subthreshold depression among university students from deteriorating into depression.

## 1. Introduction

Individuals with subthreshold depression do not meet the diagnostic criteria for depression but experience depressive symptoms, such as depressed mood and the loss of interest or pleasure [1]. The prevalence of subthreshold depression has been estimated at 29.2%, which is higher than what is reported for depression [2]. Subthreshold depression in university students is a risk factor for depression [3]. Additionally, depression during the period of university education often follows a chronic course and leads to negative outcomes, such as interpersonal difficulties, poor academic performance, and increased suicide rates [4]. Therefore, various problems are likely to be caused by subthreshold depression in university students with subthreshold depression and there is an urgent need to improve subthreshold depression in these students and to prevent it from worsening to depression. Therefore, designing effective interventions requires identifying the characteristics of subthreshold depression among university students.

In the UK, the guidelines of the National Institute for Health and Care Excellence recommend low-intensity psychological treatments for individuals with subthreshold depression [5]. One such potential treatment is behavioral activation, which identifies the context in which patients are avoiding aversive situations or experiences with a constant awareness of the situation–action–outcome and breaks the vicious cycle in life that is caused by the avoidance [6,7]. It then reveals contingency of how they maintain this state in order to break the vicious cycle, not merely to increase the number of pleasurable activities but to promote and expand their repertoire of behaviors toward desired goals or values [6,7]. Simple and easy-to-implement behavioral activation is an empirically supported intervention against depression [8,9,10,11,12,13,14]. It is regarded as an effective psychotherapy for both adults and young people with depression [15,16,17,18]. A previous study has reported that the main behavioral characteristic of an adolescent with subthreshold depression is the low frequency of positive environmental reinforcement [19]. One of the therapeutic components of behavioral activation is to promote a repertoire of behaviors toward desired goals or values and to help people access positive environmental reinforcement in their everyday lives [6,7]. Therefore, behavioral activation may be an important treatment for individuals with subthreshold depression. In an intervention study, behavioral activation has been demonstrated to be effective for university students with moderate depressive symptoms and for those who meet the criteria for subthreshold depression [16,20]. In a study of behavioral activation among university students, a one-year follow-up showed a significant difference in depressive symptoms between the intervention and control groups; however, the effect size decreased from the point of intervention to one year later and the intervention did not show a significant effect on preventing the onset of depression [21]. Furthermore, although a meta-analysis has shown the effect of psychotherapy on subthreshold depression before and after treatment, its effectiveness in preventing the onset of depression in adolescents remains controversial [22]. Similarly, another study found behavioral activation targeting subthreshold depression to be effective before and after the treatment [16]. However, one of the challenges to the prevention of the onset of depression is that current behavioral activation approaches to subthreshold depression may be overlooking some crucial factors. To identify these factors, it is necessary to clarify differences, if any, in the mechanisms of behavioral activation among healthy university students and those with depression or subthreshold depression. If differences in the mechanism-related factors of behavioral activation depend on the degree of depressive symptoms, an appropriate approach should be tailored to individuals’ states of depressive symptoms.

In avoidance of depression, as Ferster suggested, many activities seen in individuals with depression are characterized by their escape from and avoidance of aversive life experiences and a concomitantly reduced frequency of positively reinforced behavior [23]. Avoidance not only alleviates individuals’ distress in the short term but can also increase their depressive symptoms in the long term [7]. Avoidance patterns may be risk factors for depression [24]. Quigley et al. found that participants with depression reported higher levels of avoidance than remitted participants with depression, who, in turn, reported higher levels of avoidance than non-clinical participants [25]. Their study suggests that avoidance cannot be conceptualized as merely a consequence of, or response to, current depressive symptomatology but may be a vulnerability factor for depression, which persists in an attenuated form outside active depressive episodes and maintains the risk for future episodes. Moreover, a longitudinal study on university students with subthreshold depression revealed that those who met the criteria for subthreshold depression to depression after one year presented no significant change in either goal-directed activity or reinforcement scores but reported significantly higher avoidance scores [26]. Based on the results of these studies, focusing on avoidance as a factor associated with individuals’ deterioration from subthreshold depression to depression may be necessary. Four patterns of avoidance have been identified, namely behavioral–social, behavioral–nonsocial, cognitive–social, and cognitive–nonsocial avoidance [27]. A comparison between healthy participants and those with depression revealed that the latter had a significantly higher frequency of avoidance than the former [28]. However, there are few studies on avoidance among university students with subthreshold depression and more research is needed on the relationship between subthreshold depression and avoidance.

**Figure 1:**
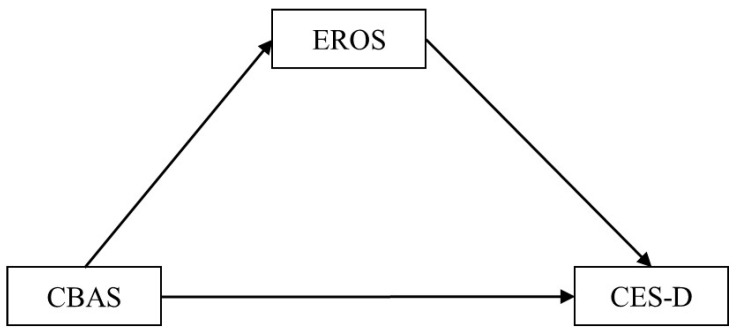
Hypothetical mediation model. Notes: CBAS, Cognitive–Behavioral Avoidance Scale; EROS, Environmental Reward Observation Scale; CES-D, Center for Epidemiologic Studies Depression Scale.

For healthy university students, studies provide support for positive environmental reinforcement as a significant mediator between the four patterns of avoidance and depression and further highlight the relevance of avoidance and positive environmental reinforcement in behavioral conceptualizations of depression [29]. However, these studies have rarely examined university students with subthreshold depression or depression. Moreover, although the four patterns of avoidance have been identified, it is unclear whether the findings reported on university students with subthreshold depression or depression are similar to those reported on their healthy counterparts. Additionally, research on the behavioral activation mechanism is still lacking [30,31]. Further research is, therefore, needed to theorize the role of avoidance in depression based on the behavioral-activation theory. Based on the findings of previous studies [6,23,29], positive environmental reinforcement is a mediating factor between avoidance and depressive symptoms (Figure 1). If we could clarify the relationship between the four avoidance patterns, positive environmental reinforcement, and depressive symptoms in university students who fit the criteria for subthreshold depression or depression, we could provide basic data on the role of avoidance in university students with subthreshold depression. Therefore, this study used mediation analysis to identify associations between the four avoidance patterns, positive environmental reinforcement, and depressive symptoms in healthy university students and those who fit the criteria for subthreshold depression or depression.

Avoidance is considered a precursor to the reduction in positive reinforcement [6,23]. Avoidance exacerbates depressive symptoms [7]. The study is based on two hypotheses. (1) All three groups of healthy university students as well as university students with subthreshold depression and those with depression show negative associations with four avoidance patterns and reward perception. (2) The four avoidance patterns will be positively associated with depressive symptoms in all three groups of university students.

The prevalence of subthreshold depression is higher than that reported for depression [2]. Subthreshold depression in university students is a risk factor for depression [3]. Avoidant behavior is also thought to be a factor in the deterioration of subthreshold depression to depression [26] but few studies have examined the characteristics of avoidant behavior exhibited by university students with subthreshold depression and depression. Conducting this study will provide new basic knowledge to prevent subthreshold depression from deteriorating into depression.

## 2. Materials and Methods

### 2.1. Participants

A total of 1200 Japanese undergraduate students (600 women and 600 men) were recruited from Rakuten Insight, Inc., a market research company in Japan that conducts online surveys (Tokyo, Japan). The participants of this study were university students aged 18–24 who enrolled in universities throughout Japan; who read the written explanation regarding their consent to participate in the study; and who gave their consent by clicking on a button signifying agreement before responding to the questionnaire. The average age of the participants was 20.61 years (SD = 1.59), ranging from 18 to 24 years.

### 2.2. Procedure

The study protocol was reviewed and approved by the Ethics Committee of the first author’s institution. Additionally, all study procedures were performed according to the relevant laws and institutional guidelines. The study procedure was explained to the participants using a slide presentation after their recruitment. Next, participants were asked to read an explanation of the study and provide informed consent by clicking on a button signifying agreement before responding to the questionnaire. Only those who agreed to participate responded to the questionnaire.

### 2.3. Measures

#### 2.3.1. Center for Epidemiologic Studies Depression Scale

Radloff developed the original Center for Epidemiologic Studies Depression Scale (CES-D) [32]. The original CES-D comprises 20 items scored on a 4-point scale, ranging from 1 (rarely or never [less than 1 day]) to 4 (most or all the time [5–7 days]) [32]. The original CES-D has good reliability and validity [32]. This study used the Japanese version of the CES-D to measure depressive symptoms in the participants. This Japanese version of the CES-D has good reliability and validity [33]. Cronbach’s alpha for the CES-D was 0.92 in this study.

#### 2.3.2. Patient Health Questionnaire

The original version of the Patient Health Questionnaire (PHQ-9) consists of nine items rated on a 4-point Likert scale, ranging from 0 (not at all) to 3 (nearly every day) [34]. The original PHQ-9 has good reliability and validity [34]. This study used the Japanese version to measure depressive symptoms in the participants. The Japanese version of the PHQ-9 has good reliability and validity [35]. Cronbach’s alpha for the PHQ-9 was 0.87 in this study.

#### 2.3.3. Cognitive–Behavioral Avoidance Scale

Ottenbreit and Dobson16 developed the Cognitive–Behavioral Avoidance Scale (CBAS) [27]. The original CBAS comprises 31 items scored on a 5-point scale, ranging from 1 (not at all true for me) to 5 (extremely true for me) [27]. Its four subscales include the Cognitive–Behavioral Avoidance Scale–Behavioral Social (CBAS-BS), Cognitive Social (CBAS-CS), Behavioral Nonsocial (CBAS-BN), and Cognitive Nonsocial (CBAS-CN). The original CBAS has good reliability and validity [27]. This study used the Japanese version of the CBAS to measure depressive symptoms, which also showed good reliability and validity [36]. Cronbach’s alphas for CBAS-BS, CBAS-CS, CBAS-BN, and CBAS-CN were 0.88, 0.82, 0.88, and 0.89, respectively, in this study.

#### 2.3.4. Environmental Reward Observation Scale

Armento and Hopko developed the original Environmental Reward Observation Scale (EROS) [37]. The original EROS comprises 10 items scored on a 4-point Likert-type scale, ranging from 1 (strongly disagree) to 4 (strongly agree) [37]. The original EROS has good reliability and validity [37]. This study used the Japanese version of the EROS, which also showed good reliability and validity, to measure the participants’ exposure to environmental rewards deemed necessary to increase response-contingent positive environmental reinforcement. This version of the EROS has good reliability and validity [38]. Cronbach’s alpha for the EROS was 0.76 in this study.

### 2.4. Definitions of Participating Groups

In previous research, subthreshold depression includes clinically significant depressive symptoms that do not meet the diagnostic criteria for Major Depressive Disorder (MDD) [1,39]. The doctor’s diagnosis and a structured interview are the best ways to show that patients do not fit the criteria for MDD. However, because this study was based on an internet survey, a doctor’s diagnosis and a structured interview could not be conducted. Additionally, the PHQ-9 has been shown to be a convenient tool to screen for MDD [40]. Therefore, we decided to use the PHQ-9 and CES-D in this study to identify those with subthreshold depression. Depression and subthreshold depression were defined based on the following criteria. The depressed group comprised participants who always included an item from the PHQ-9 based on the fifth edition of the Diagnostic and Statistical Manual of Mental Disorders (DSM-V) algorithm diagnosis, for example, “Little interest or pleasure in doing things” or “Feeling down, depressed, or hopeless”, answered more than five of the PHQ-9 items with the response, “more than half of the days”. Note that in the diagnosis of MDD, the ninth item (PHQ-9), which is related to suicide, is evaluated by counting it as one, even if the respondent answered several days. The subthreshold depression group included those who did not meet the DSM-V criteria for major depressive disorder using the PHQ-9 described above and had a CES-D score of 16 or higher. Finally, the healthy group included those who did not meet the DSM-V criteria for major depressive disorder and subthreshold depression.

### 2.5. Data Analyses

We used SPSS version 28.0 (SPSS Japan Inc., Tokyo, Japan) and Mplus version 7.3 (Muthen and Muthen, Los Angeles, CA, USA) to analyze our data. First, the CES-D, EROS, CBAS-BS, CBAS-BN, CBAS-CS, and CBAS-CN were investigated using a one-way analysis of variance (ANOVA). Next, we conducted a Pearson’s correlation analysis and mediation analysis with bootstrapping to clarify the role of positive reinforcers when avoidance induces depressive symptoms. A previous study reported that the bias-corrected (BC) interval provides the most accurate confidence and highest statistical power and should thus be the method of choice if it is feasible to conduct resampling [41]. Therefore, we used the BC method to examine the confidence intervals (CI). In the bootstrapping procedure, if the 95% BC CI does not contain zero, it is considered statistically significant at the 5% level. Therefore, the BC 95% CI was used to test for significance at the 5% level. The BC 95% CI was calculated based on 5000 bootstrap samples from a previous study [29].

## 3. Results

### 3.1. Participant Characteristics

Participants were allocated to the healthy group (*n* = 746; mean age: 20.61; male: 387; female: 359), subthreshold-depression group (*n* = 308; mean age: 20.59; male: 140; female: 168), and depression group (*n* = 146; mean age: 20.71; male: 73; female: 73) based on the CES-D and PHQ-9. The results showed that 25.66% of university students met the criteria for subthreshold depression.

### 3.2. Descriptive Statistics and Correlation Analysis

In this study, one-way ANOVA yielded significant differences in interaction effects on the CES-D [F(2,1197) = 1503.28, *p* < 0.01], EROS [F(2,1197) = 195.05, *p* < 0.01], CBAS-BS [F(2,1197) = 121.02, *p* < 0.01], CBAS-BN [F(2,1197) = 115.01, *p* < 0.01], CBAS-CS [F(2,1197) = 96.21, *p* < 0.01], and CBAS-CN [F(2,1197) = 117.72, *p* < 0.01]. Next, we conducted multiple comparisons of the CES-D, EROS, CBAS-BS, CBAS-BN, CBAS-CS, and CBAS-CN scores. For the CES-D, CBAS-BS, CBAS-BN, CBAS-CS, and CBAS-CN scores, there were significant differences among the three groups; the depression group had the highest score. Similarly, there were significant differences in the EROS scores among the three groups; however, in this case, the healthy group had the highest score (Table 1).

Next, we applied a correlation analysis to evaluate the relationship between avoidance, positive environmental reinforcement, and depressive symptoms. In the depression group, the EROS score was negatively correlated with CBAS-BN (r = −0.29, *p* < 0.01) and CES-D scores (r = −0.54, *p* < 0.01). In the subthreshold-depression group, the EROS score was negatively correlated with CBAS-BS (r = −0.14, *p* < 0.05), CBAS-BN (r = −0.21, *p* < 0.01), and CES-D scores (r = −0.21, *p* < 0.01). In the healthy group, the EROS score was negatively correlated with CBAS-BS (r = −0.35, *p* < 0.01), CBAS-BN (r = −0.36, *p* < 0.01), CBAS-CS (r = −0.35, *p* < 0.01), CBAS-CN (r = −0.40, *p* < 0.01), and CES-D scores (r = −0.40, *p* < 0.01).

### 3.3. Mediation Analysis

We conducted mediation analysis using the bootstrapping method with CES-D as the dependent variable and the subscales of CBAS as the independent variable, with EROS as a mediating variance for depression, subthreshold depression, and healthy groups (Table 2). In the depression group, CBAS-BN had a direct effect on CES-D (estimate of the direct effect: 0.457, 95% BC CI: 0.132 to 0.767), whereas EROS significantly mediated the relationship between only CBAS-BN and CES-D (estimate of the indirect effect: 0.265, 95% BC CI: 0.120 to 0.451). In the subthreshold-depression group, CBAS-BS had a direct effect on CES-D (estimate of the direct effect: 0.128, 95% BC CI: 0.010 to 0.238), whereas EROS significantly mediated the relationship between CBAS-BS and CES-D (estimate of the indirect effect: 0.026, 95% BC CI: 0.004 to 0.068). Additionally, CBAS-BN had a direct effect on CES-D (estimate of the direct effect: 0.176, 95% BC CI: 0.020 to 0.336), whereas EROS significantly mediated the relationship between CBAS-BN and CES-D (estimate of the indirect effect: 0.052, 95% BC CI: 0.015 to 0.110). In the healthy group, EROS mediated the relationship between the four avoidance patterns and CES-D.

## 4. Discussion

This study used mediation analysis to investigate associations between the four avoidance patterns, positive environmental reinforcement, and depressive symptoms among healthy university students and those with subthreshold depression or depression. The results of the mediation analysis indicated that the four avoidance patterns had a significant indirect relationship with depressive symptoms through positive environmental reinforcement, indicating that positive environmental reinforcement mediated the relationship between the four avoidance patterns and depressive symptoms in the healthy group. However, in the subthreshold-depression group, positive environmental reinforcement was mediated by behavioral social and behavioral non-social avoidance and depressive symptoms. In the group of participants with depression, positive environmental reinforcement was mediated only by behavioral nonsocial avoidance and depressive symptoms. Our results indicate different associations between avoidance, positive environmental reinforcement, and depressive symptoms in the groups with depression, subthreshold depression, and healthy conditions.

In this study, the PHQ-9 and CES-D were used to divide participants into three groups: depression, subthreshold depression, and healthy conditions. Of the total participants, 12.17% fit the depression group, 25.66% fit the subthreshold-depression group, and the rest fit the healthy group. A previous study reported a 12-month depression prevalence rate of 18.5% among university students [42]. As the prevalence of subthreshold depression is thought to be approximately 29.2%, the ratio of depression to subthreshold depression shown in this study is likely to be similar to that in previous studies. Our study found that, among the three groups, the group with depression had significantly higher scores on all four avoidance patterns, which is consistent with research showing a positive relationship between avoidance and depression [27,43,44]. This suggests that avoidance may be an important intervention target for university students with subthreshold depression.

The results of our mediation analysis showed a positive environmental reinforcement mediating the relationship between the four avoidance patterns and depressive symptoms but only in the healthy group. This result is consistent with that of previous studies [29]. However, in the group with depression, positive environmental reinforcement only mediated the relationship between nonsocial behavioral avoidance patterns and depressive symptoms. Furthermore, in the group with subthreshold depression, positive environmental reinforcement only mediated the relationship between behavioral avoidance patterns (social and nonsocial) and depressive symptoms. Behavioral avoidance has been shown to be related to depressive symptoms, mediated by reduced positive reinforcement [45]. These results were also consistent with those of previous studies in groups with depression and subthreshold depression. Additionally, this study showed that the relationship between avoidance, positive environmental reinforcement, and depressive symptoms differs according to the degree of depressive symptoms. To the best of our knowledge, no previous study has reported results similar to those of our study.

In previous studies, avoidance was considered a precursor to the reduction in positive reinforcements [6,23] and avoidance exacerbates depressive symptoms [7]. Therefore, the research hypothesis of the present study was that the above-mentioned relationship would be demonstrated in the three groups. However, the results of the present study showed that only the healthy group demonstrated this relationship as stated in the hypothesis. University students with subthreshold depression and depression showed no significant relationship between cognitive avoidance and positive environmental reinforcement. Previous studies on depression have shown an association between behavioral avoidance and positive environmental reinforcement, while no association was found between rumination and positive environmental reinforcement [45]. The results of this study and the previous study [45] are somewhat consistent. However, this previous study did not examine behavioral avoidance separately in social and nonsocial situations [45]. In the present study, both social and nonsocial situations were shown to be mediated with positive environmental reinforcement in subthreshold depression but only nonsocial situations were shown to be mediated with depression. It has been noted that extensive avoidance may result in further loss of positive environmental reinforcement [46]. Furthermore, rigid avoidance vastly narrows the range of behavioral options available and will most likely result in stable reductions in response-contingent social reinforcement [47]. Moreover, a study has suggested that avoidance blocks opportunities to restore important sources of positive environmental reinforcement [48]. Considering our results regarding the difference between sub-threshold depression and depression, the present study supports the idea that the association between avoidance behavior and social reinforcement decreases with the frequency of avoidance behavior. However, the present study does not clarify whether positive environmental reinforcement is associated with behavioral avoidance or why the association between cognitive avoidance and positive environmental reinforcement is blocked in the subthreshold depression and depression groups. Further research, such as longitudinal studies, on this topic is required.

Moreover, the present study showed a direct association between avoidance and depressive symptoms in the groups with depression, subthreshold depression, and healthy conditions. This result was consistent with our research hypothesis. Studies have shown that avoidance elicits functional impairment and is associated with life stressors [49]. Martell’s behavioral activation model specifically emphasizes the role of avoidant behaviors [6,50]. In this model, which is consistent with traditional behavioral models, behavioral activation modifies a person’s environment through behavioral change, which in turn increases access to positively reinforcing events and activities [7]. Thus, the four avoidance patterns are intervention targets and it is important to modify avoidance patterns into other behaviors to increase positive environmental reinforcement. Our study revealed that in the groups with depression, behavioral nonsocial avoidance was negatively associated with positive environmental reinforcement; in the subthreshold-depression group, social and nonsocial behavioral avoidance were negatively associated with positive environmental reinforcement. If behavioral activation involves increasing access to positively reinforcing events and activities, it might be necessary to intervene first with nonsocial-avoidance behaviors in depression and behavioral avoidance in social and nonsocial situations in subthreshold depression. However, this study did not implement behavioral activation or demonstrate its relevance; therefore, further research is necessary on these issues.

Finally, it is important to note the following limitations of this study. First, as this study used a large group of university students, the survey was conducted online. Therefore, the PHQ-9 and CES-D, rather than a clinical evaluation by physicians, were used to divide the participants into groups with depression, subthreshold depression, and healthy conditions. Future studies might need to be conducted using physician consultations and structured interviews to achieve greater diagnostic accuracy. Second, as this study aimed to examine the characteristics of university students with subthreshold depression, it was conducted using a sample only of university students. In the future, it will be necessary to examine whether the same results can be obtained if the general population is targeted. Third, this study aimed to examine associations among the four avoidance patterns, positive environmental reinforcement, and depressive symptoms using mediation analysis. Causal relationships must therefore be interpreted with caution; we cannot conclude conclusively whether increased avoidance leads to a decrease in the frequency of positive environmental reinforcement. Therefore, future longitudinal studies are warranted. Fourth, this study did not examine gender differences; gender differences should also be examined to clarify the characteristics of university students. However, including consideration of sex differences would double the number of each group and raise concerns about test power. We believe that future research should examine sex differences with a larger sample.

## 5. Conclusions

This study used mediation analysis to identify associations between the four avoidance patterns, positive environmental reinforcement, and depressive symptoms in healthy university students and those who fit the criteria for subthreshold depression or depression. The present study demonstrates that associations among avoidance patterns, positive environmental reinforcement, and depressive symptoms vary according to the degree of depressive symptoms. Previous studies suggested that proactive management of subthreshold depression could be effective in managing the increasing prevalence of major depression [51]. The results revealed by this study provide new foundational insights to prevent subthreshold depression from university students deteriorating to depression.

## Figures and Tables

**Table 1:** Results of descriptive data between three groups.

	Depression- Group	Subthreshold-Depression Group	Healthy Group		
	*N* = 146 (12.17%)	*N* = 308 (25.66%)	*N* = 746 (62.17%)	*p*-value	Multiple comparison
Results of one-way ANOVA					
CES-D	32.03 (9.03) ^1^	23.09 (5.89) ^2^	10.44 (3.22) ^3^	<0.01	3 < 2 < 1
EROS	21.26 (4.96) ^1^	23.44 (3.83) ^2^	27.55 (4.25) ^3^	<0.01	1 < 2 < 3
CBAS-BS	25.74 (6.72) ^1^	23.19 (6.01) ^2^	17.97 (7.00) ^3^	<0.01	3 < 2 < 1
CBAS-BN	19.91 (4.59) ^1^	18.49 (4.34) ^2^	14.77 (4.89) ^3^	<0.01	3 < 2 < 1
CBAS-CS	20.73 (5.89) ^1^	18.90 (5.54) ^2^	14.76 (5.99) ^3^	<0.01	3 < 2 < 1
CBAS-CN	30.35 (8.07) ^1^	27.67 (6.95) ^2^	21.68 (7.92) ^3^	<0.01	3 < 2 < 1

Note: Values in parentheses represent standard deviation. There is a significant difference between different numbers (1 < 2 < 3: *p* < 0.01, 3 < 2 < 1: *p* < 0.05). CES-D, Center for Epidemiologic Studies Depression Scale; EROS, Environmental Reward Observation Scale; CBAS-BS, Cognitive–Behavioral Avoidance Scale–Behavioral Social; CBAS-BN, Cognitive–Behavioral Avoidance Scale-Behavioral Nonsocial; CBAS-CS, Cognitive–Behavioral Avoidance Scale–Cognitive Social; CBAS-CN.

**Table 2:** Results of mediation analysis.

	Estimate	BC 95% Cl		Estimate	BC 95% Cl		Estimate	BC 95% Cl		Estimate	BC 95% Cl	
	CBAS-BS → EROS (a)			EROS → CES-D (b)			CBAS-BS → CES-D (c)			Indirect (a*b)		
Depression	−0.110	−0.233	0.017	−0.925	−1.162	−0.671	0.251	0.039	0.444	0.101	−0.012	0.226
Subthreshold depression	−0.089	−0.167	−0.013	−0.296	−0.471	−0.100	0.128	0.010	0.238	0.026	0.004	0.068
healthy	−0.214	−0.256	−0.175	−0.250	−0.311	−0.190	0.090	0.061	0.120	0.054	0.039	0.071
	CBAS-BN → EROS (a)			EROS → CES-D (b)			CBAS-BN → CES-D (c)			Indirect (a*b)		
Depression	−0.310	−0.479	−0.131	−0.854	−1.106	−0.585	0.457	0.132	0.767	0.265	0.120	0.451
Subthreshold depression	−0.183	−0.281	−0.082	−0.283	−0.463	−0.082	0.176	0.020	0.336	0.052	0.015	0.110
healthy	−0.314	−0.375	−0.249	−0.263	−0.323	−0.202	0.095	0.050	0.141	0.082	0.061	0.108
	CBAS-CS → EROS (a)			EROS → CES-D (b)			CBAS-CS → CES-D (c)			Indirect (a*b)		
Depression	0.022	−0.160	0.174	−0.985	−1.216	−0.743	0.283	0.037	0.513	−0.022	−0.178	0.116
Subthreshold depression	−0.015	−0.102	0.072	−0.317	−0.483	−0.127	0.207	0.078	0.324	0.005	−0.017	0.040
healthy	−0.250	−0.299	−0.204	−0.249	−0.310	−0.188	0.107	0.074	0.142	0.062	0.045	0.082
	CBAS-CN → EROS (a)			EROS → CES-D (b)			CBAS-CN → CES-D (c)			Indirect (a*b)		
Depression	−0.094	−0.198	0.019	−0.923	−1.159	−0.669	0.213	0.034	0.387	0.087	−0.013	0.191
Subthreshold depression	−0.032	−0.098	0.031	−0.307	−0.477	−0.115	0.157	0.063	0.247	0.010	−0.007	0.039
healthy	−0.216	−0.251	−0.182	−0.230	−0.292	−0.169	0.096	0.069	0.123	0.050	0.036	0.066

Note: CBAS-BS, Cognitive–Behavioral Avoidance Scale–Behavioral Social; CBAS-BN, Cognitive–Behavioral Avoidance Scale-Behavioral Nonsocial; CBAS-CS, Cognitive–Behavioral Avoidance Scale–Cognitive Social; CBAS-CN; EROS, Environmental Reward Observation Scale; BC 95% CI = bias-corrected 95% confidence interval.

## Data Availability

The datasets generated for this study will not be made publicly available as the authors do not have permission to share the data.
